# Analysis of the Climate Impact on Occupational Health and Safety Using Heat Stress Indexes

**DOI:** 10.3390/ijerph22010130

**Published:** 2025-01-20

**Authors:** Guilherme Neto Ferrari, Guilherme Custódio dos Santos, Paulo Cesar Ossani, Gislaine Camila Lapasini Leal, Edwin Vladimir Cardoza Galdamez

**Affiliations:** 1 Computer Science Postgraduate Program, Informatics Department, State University of Maringá, Av. Colombo, 5790, Bloco C56, Zona 7, Maringá 87020-900, Paraná, Brazil; gclleal@uem.br; 2 Production Engineering Department, State University of Maringá, Av. Colombo, 5790, Bloco 19/20, Zona 7, Maringá 87020-900, Paraná, Brazil; ra118433@uem.br; 3 Statistics Department, State University of Maringá, Av. Colombo, 5790, Bloco E90, Zona 7, Maringá 87020-900, Paraná, Brazil; pcossani2@uem.br; 4 Production Engineering Postgraduate Program, Production Engineering Department, State University of Maringá, Av. Colombo, 5790, Bloco 19/20, Zona 7, Maringá 87020-900, Paraná, Brazil; evcgaldamez@uem.br; 5 Applied Social Sciences Center, Accounting Sciences Postgraduate Program, State University of Maringá, Av. Colombo, 5790, Bloco B12, Zona 7, Maringá 87020-900, Paraná, Brazil

**Keywords:** occupational health and safety, heat stress, heat stress index, multiple correspondence analysis

## Abstract

Workers may be exposed to conditions that put their physical and mental integrity at risk, from workplace settings to climate characteristics. Heat stress is a harmful health condition caused by exceeding the human body’s tolerance limits, leading to illness and increasing the chance of work accidents. Heat stress indexes, such as the Humidex and the Heat Index (HI), are used to measure these impacts. These indexes are significant as they provide a quantitative measure of the heat stress experienced by workers, taking into account both environmental and individual factors. Objective: This study aims to compare multiple heat stress indexes, relating them to historical Brazilian occupational accident data. Methods: We selected eight indexes and applied multiple correspondence analysis to each one, a statistical method that generates graphs to visualize the association between variables in a database. Results: The analysis and comparison of the graphs indicated that seven of the eight indexes presented similar behavior. It was also possible to relate ranges of index values with specific characteristics of the accidents. Conclusions: The technique allowed us to analyze the relationship between climate and work accidents and showed that the choice of the heat stress index does not significantly alter the results for most indexes studied.

## 1. Introduction

Neglecting the health and safety of workers leads to an increase in occupational injuries and diseases, directly impacting a company’s operational performance and resulting in economic losses [1]. In Brazil, over BRL 300 million was spent on compensations for occupational accidents [2]. Multiple variables influence the occurrence of workplace accidents, from the repetitive nature of the activity to the lack of use of protective equipment [3]. Specific field-oriented and outdoor industries are also susceptible to environmental variables that may affect the workplace comfort and well-being of workers; construction, agriculture, and forestry are a few examples of these cases, where the worker is exposed to hours of intensive physical tasks in a high ambient temperature, leading to heat stress [4]. Heat stress is the sum of the heat load an individual may be exposed to from the combination of metabolic and environmental heat. The body should dissipate the heat; however, strenuous physical activities, high ambient temperature, and clothing can affect heat release, making the body retain more than it should, increasing health-related risks, such as heat-related injuries [5,6]. When dealing with heat stress, an approach that cab be used is to assess the workers’ body responses to such thermal stress, which is called heat strain, i.e., the physiological response to help dissipate the heat, which can be perceived with regard to the changes in heart rate and core and skin temperature [7,8]. Failing to maintain a body temperature below its tolerance limits can lead to serious health problems in the short and long term [9,10].

Several studies have proposed methods to evaluate the effects of high temperatures on occupational settings. When it comes to assessing heat stress, most methods collect environmental variables of the workplace, such as Heat Index and Humidex, taking into consideration air temperature and relative humidity [7]. However, these approaches are criticized as they generalize the effects of these environmental factors on every worker in that setting and do not consider the different reactions that different bodies may have. Therefore, a more specific approach to reflect each worker’s characteristics and body responses to heat stress is needed [11]. Approaches such as Personal Heat Stress (PHS) can overcome this limitation by considering environmental and individual factors such as clothing and metabolic rate [12,13]. Although this approach can be more accurate in representing the physical responses of individuals to heat stress, its application requires direct access to workers’ data. The means of gathering these data might require the use of invasive equipment, such as rectal or esophageal probes [14], expensive techniques such as gastrointestinal pills, or wearable devices that monitor real-time data [15,16].

When it comes to analyzing the historical records of occupational accidents or illnesses, the research relies on available databases, such as records of workers’ compensation claims [17,18], national occupational health databases [19], or national statistics reports [20]. In this approach, access to detailed worker data is complicated and dependent on the source of the information; most of the time, this type of database gathers information regarding the whole country or industry; therefore, the data are anonymized. These databases include data such as the date and place of the incident, demographic information about the worker, and medical aspects of what happened, such as the International Classification of Diseases (ICD) of the injury or body parts affected [21], which means that using personal worker data to analyze large amounts of data to investigate occupational heat stress is not frequent in the literature. Therefore, this type of study cannot use personal factors such as metabolic rate and clothing.

The calculation of heat stress can be made through the use of indexes, which are used to represent the risk in ranges of tolerance. Different indexes in the literature differ regarding the climate variables they consider in the calculation, the formulation of the equation to estimate the risk, and the tolerance defined [22]. Some heat indexes are more frequently used, such as the WBGT, the most popular and accepted index, included in national and international standards [22,23].

Estimated indexes calculated from climate variables are used to represent this risk. In the occupational context, using indexes allows us to assess the influence of a climate on OSH. Different indexes have been proposed over the decades in the literature [24,25,26]. Focusing on indexes that only use environmental factors, one of the first ones developed was the Effective Temperature Index, which analyzes heat stress based on temperature and relative humidity [27]. In 1945, the effects of wind in cold environments were being considered, which led to the Wind Chill Temperature Index, developed to consider the effects of cold, used mainly during winter. Its equation includes air temperature and wind speed [26,28].

The Discomfort Index was proposed in 1959 and includes relative humidity and air temperature to identify thermal discomfort [29]. The Humidex was developed in 1979 and considers air temperature and relative humidity [30]. Apparent Temperature (AT) was developed in 1984 and initially applied in the US and Australia, measuring air temperature and relative humidity [31]. Heat Index was created based on AT in 1990 and includes air temperature and relative humidity in its equation as well [32]. It is highly used by the National Oceanic and Atmospheric Administration (NOAA), which recommends it being used for temperatures below 27 °C and relative humidity below 40% [33]. Effective temperature according to the wind is based on the Effective Temperature Index; however, it differs by including the wind in its equation [34]. Finally, the Modified Discomfort Index is an alternative developed in 1998 that uses air temperature and a wet-bulb thermometer in its calculation [35].

This study aims to compare some heat stress indexes found in the literature that only use environmental factors. The comparison will be made regarding their relationship to variables that characterize the victims of occupational accidents in Brazil. This relationship will be found and represented with the application of a multiple correspondence analysis (MCA), which generates a bidimensional graph to illustrate the proximity of different attributes of a database visually. Previous studies have been able to relate heat stress index to occupational accident records using MCA and identified a few behaviors and tendencies within the data, such as a racial issue, where White people were related to lower heat stress levels. In comparison, Pardo people were related to higher and more dangerous levels of heat stress and fatal accidents [21]. We aim to replicate this approach using the same database of work accidents recorded in Brazil’s Notifiable Injury Information System (Sinan) between 2006 and 2023. However, instead of using the WBGT, we apply the same approach to eight indexes. This way, we investigate if the selection of an index is a significant factor to consider when analyzing the effects of temperature and heat stress on historical records of occupational accidents.

## 2. Materials and Methods

In this paper, we will explore the effects of heat stress on the occurrence of occupational accidents in Brazil. Our database comprises records of every type of work accident notified to health facilities nationwide from 2006 to 2023. The worker data in this database refers to their age, gender, education level, and occupation; every record is anonymized and does not include any personal physiological data. Therefore, to assess heat stress, we are including only environmental factors. We gathered meteorological data from the closest weather station to the accident location, collecting hourly data on air temperature, wind speed, and related humidity. However, it is important to note that our study is limited by the lack of personal physiological data, which could provide a more comprehensive understanding of heat stress. Additionally, our findings may not be generalizable to all occupational settings, as they are based on a specific time period and geographical location.

The first and most crucial step of this paper is gathering and organizing the data. We combined occupational accidents recorded over 18 years, from 2006 to 2018, with weather information from the same period. The work accident data were collected from the Sinan database, made available by the Brazilian Ministry of Health database. The Sinan database records every notification regarding the population’s health nationwide. The system divides these data into categories, which can be accessed openly. We selected the dataset regarding occupational accidents, which considers every record in Sinan with the ICD code of Y96. The data encompass the information filled in by the medical unit that cared for the victim of the accident, including basic information such as the date, hour, and location; demographic information about their age, gender, race, level of education, and occupation; data regarding the incident, such as the type of accident, place where it happened, people involved, body parts affected; and how the case unfolded. The database has a total of 75 attributes. We selected twelve of these variables to include in our study: age, sex, race, education, region of the country, occupation, work situation, accident location, accident type, body part affected, case evolution, and whether or not the Work Accident Report (CAT) was opened.

To associate the occupational accident with heat stress, we collected weather information for the day and hour of the accident from the closest weather station. The climate data included hourly air temperature measurements, relative humidity, and wind speed. These data are collected by automatic weather stations located around the country and provided by the Brazilian Meteorological National Institute (INMET). We gathered data from all available weather stations during the same period of 2006 to 2018. Each station dataset includes information regarding their geographic location using latitude and longitude. This information is critical to associating the climate data with each occupational accident. We used a database of cities provided by the Brazilian Institute of Geography and Statistics that lists every municipality in Brazil, a code to identify it, and its latitude and longitude. Every accident record includes a city code of where it happened. Therefore, we collected the latitude and longitude of every city in the Sinan database and used it to find the closest weather station to each accident.

Once the climate data were associated with the accident records, we treated the data by eliminating null values or values that meant “ignored” in the system, which represented a low percentage of the values in the variables but did not explain anything regarding the characteristics of the accident. Most of the exclusions were due to incomplete weather information; some cases were excluded because there was no recording during that time of the accident, which could be due to the weather station being built after the accident, for example, or because the information gathered was inconsistent, such as negative values of “9999” for every variable. This processing resulted in a total of 498,538 accident records to be analyzed.

Next, the heat stress indexes were calculated. Eight indexes were selected: Effective Temperature Index (ET), Discomfort Index (DI), Heat Index (HI), Effective Temperature Index According to the Wind (ETW), Humidex Index (HX), Apparent Temperature Index (AT), Modified Discomfort Index (MDI) and Wind Chill Temperature Index (WCT). R software was used to calculate the representations of each index [36]. We calculated the eight indexes for each accident and analyzed them separately using MCA. MCA is a statistical method that creates a matrix to assess the relationship of the variables among a database; it creates a dummy variable for each attribute and investigates their influence among each other, generating a bi-dimensional graph to represent this relationship [37]. We developed an MCA for each index separately so they would not influence the results of the others. We used the MVar package of R software [38].

The last step involved analyzing and interpreting the graphic results of MCA. To facilitate comparing the indexes, we designated different geometric shapes and colors for each one in the graph. Table 1 presents the name of the index, its acronym, and the defined symbol.

We noticed that natural groups were formed when observing the graph and the position of the variables represented by dots in the plane. To explore this behavior, we created clusters based on our observations, which will guide our analysis and simplify the comparison between the indexes. We also created an acronym for each variable to reduce its name to a maximum of three letters to facilitate reading information in the MCA graph. Each variable can receive one value among a set of possibilities; the gender or sex variable, for example, was renamed as “Sx” and can be either male or female, represented as “Sx: A” or “Sx: B”, respectively. Other variables, such as occupation or body parts, have more possible values. The age variable was grouped into ranges to simplify the amount of possibilities. Table 2 presents the acronyms of every variable and its respective values.

The application was originally created in Portuguese; therefore, the acronyms used were a simplification of the words in Portuguese.

## 3. Results

**Figure 1:**
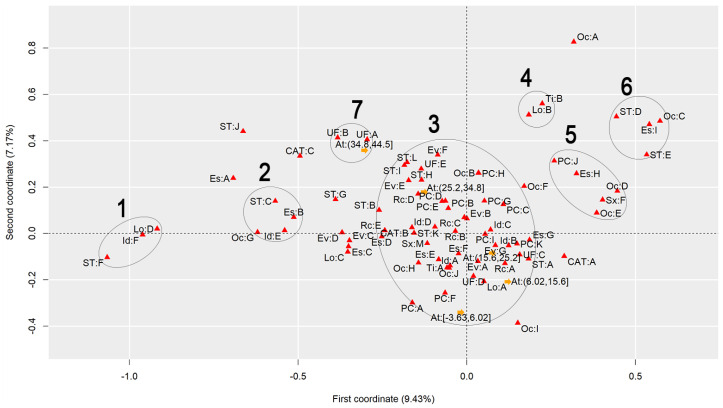
MCA graph for the Apparent Temperature Index.

**Figure 2:**
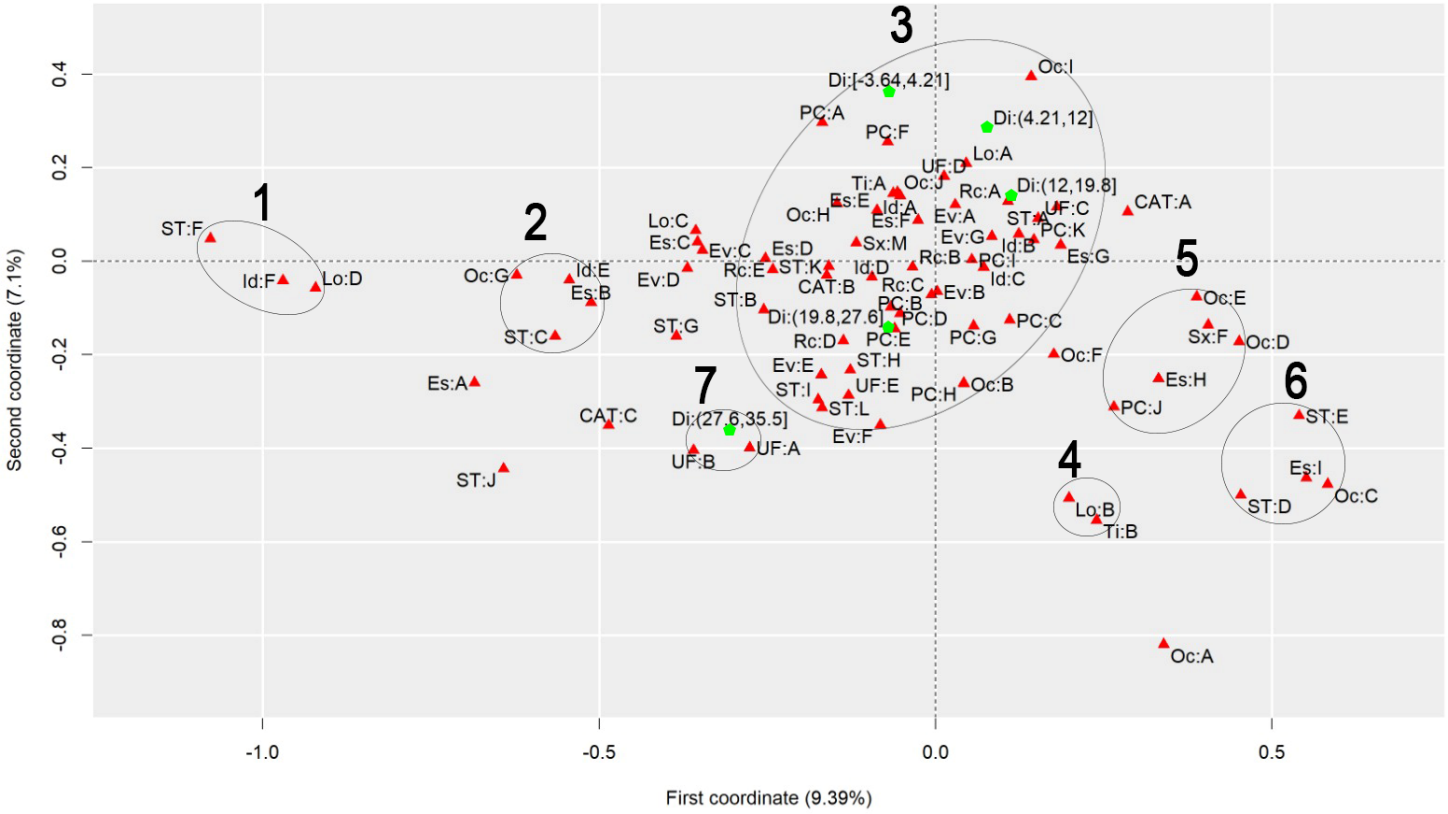
MCA graph for the Discomfort Index.

**Figure 3:**
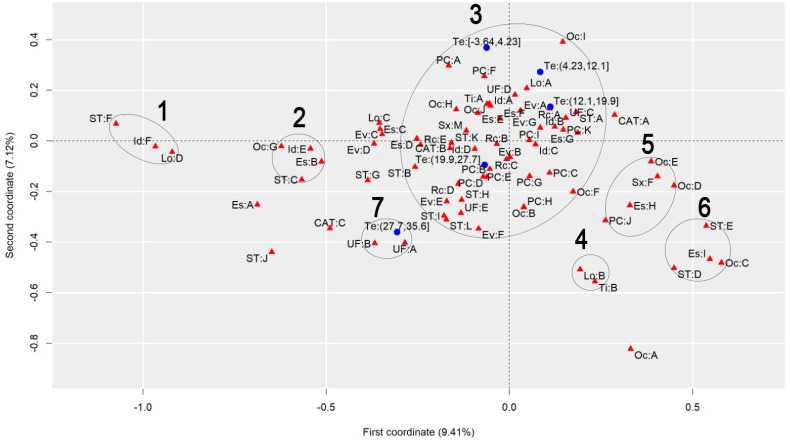
MCA graph for the Effective Temperature Index.

**Figure 4:**
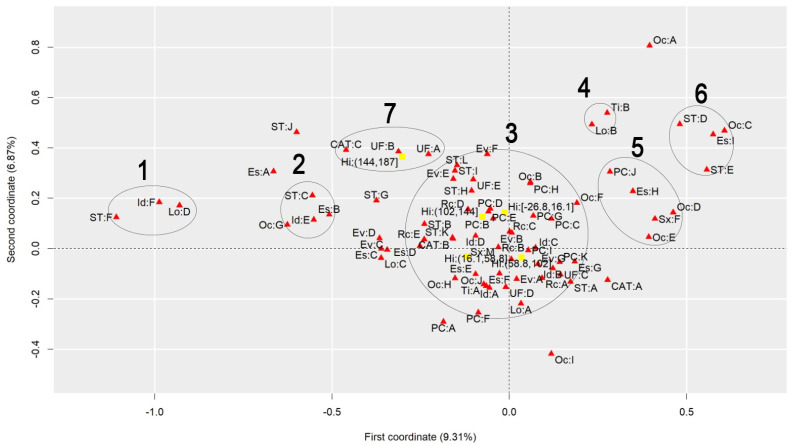
MCA graph for the Heat Index.

**Figure 5:**
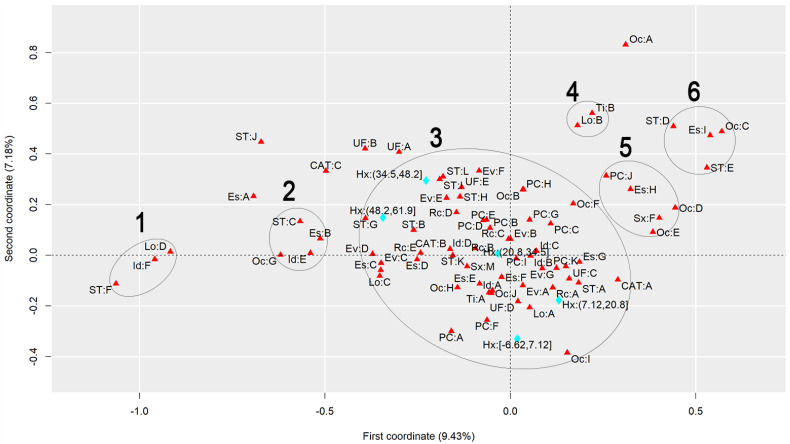
MCA graph for the Humidex Index.

**Figure 6:**
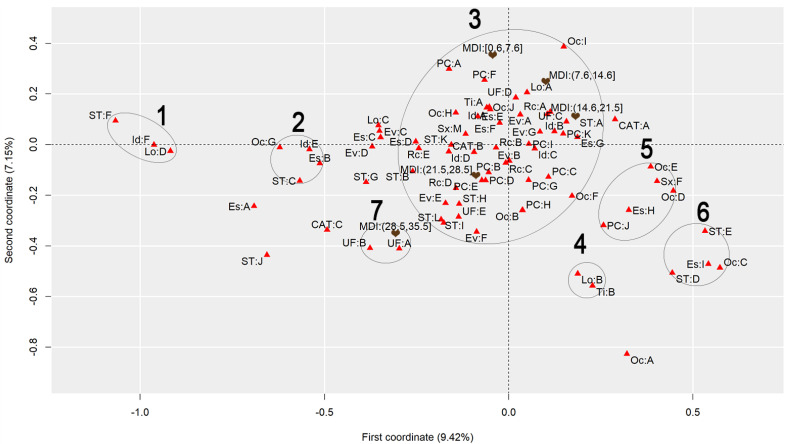
MCA graph for the Modified Discomfort Index.

**Figure 7:**
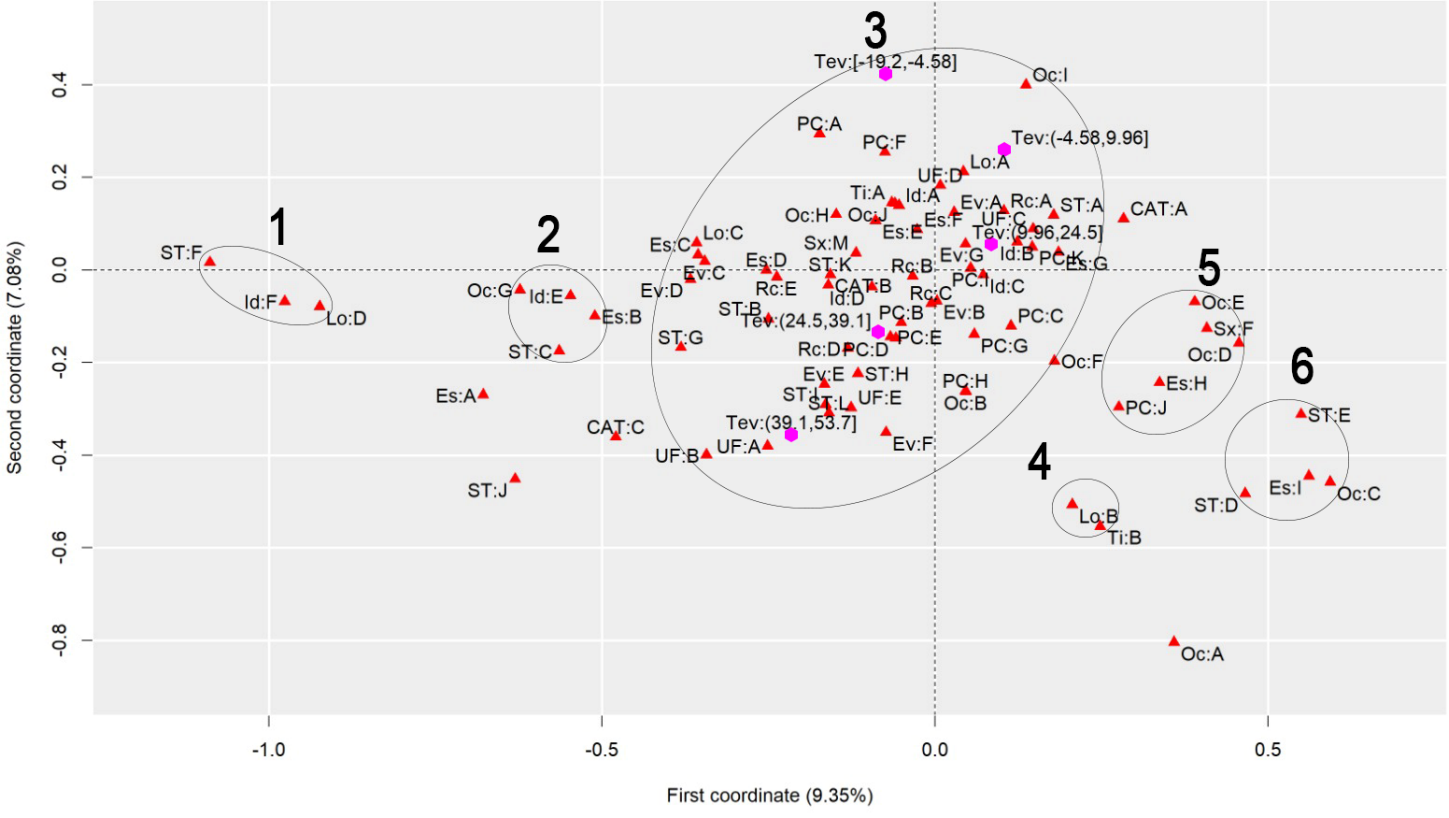
MCA graph for the Effective Temperature According to the Wind Index.

**Figure 8:**
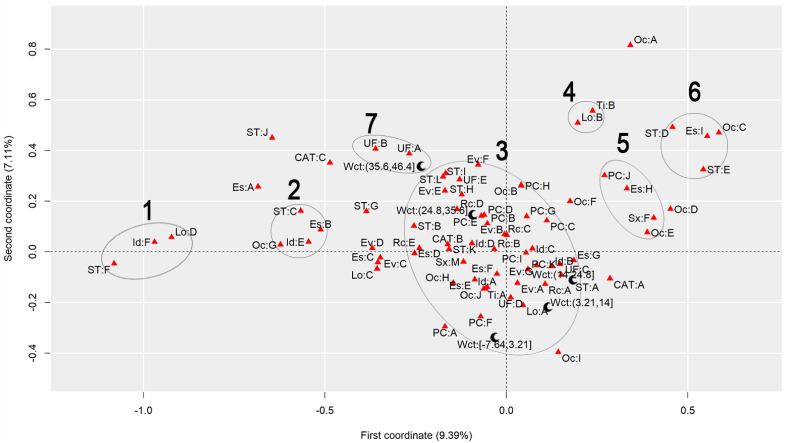
MCA graph for the Wind Chill Temperature Index.

To discuss the results regarding the comparison of different heat stress indexes, we will analyze the graphs to observe the behavior of each index, finding similarities and differences. Figure 1 presents the graph generated by applying MCA to the AT Index, Figure 2 refers to the DI, Figure 3 to the ET Index, Figure 4 to the HI, Figure 5 to the HX, Figure 6 to the MDI, Figure 7 to the ETW, and Figure 8 to the WCT. Each graph includes the delimited and numbered clusters with the specific symbols and colors of the indexes being different.

At first glance, a common pattern emerges among the generated images. The HX and ETW indexes exhibit 6 clusters, while the others show one additional cluster. Clusters 1, 2, 4, 5, and 6 are consistent across all images. The main points of divergence between the indexes are Cluster 3 and the presence or absence of Cluster 7. We will first delve into the shared clusters among the indexes, paving the way for a discussion on the unique ones. Table 3 details the attributes in Clusters 1, 2, 4, 5, and 6, and their respective values.

Some clusters indicate relationships between variables easily associated with each other, such as Cluster 1, which associates elderly and retired workers who had accidents in their own homes, or Cluster 4, which indicates a relationship between commuting accidents and public roads. Commuting accidents occur on the way between the worker’s home to work or from work to their home, and they frequently occur on public roads. These are simple associations to interpret; it is to be expected that an elderly and retired worker would be injured in their or her own home, just as it is common for commuting accidents to occur on public roads.

However, the other clusters present relationships that are not immediately apparent. For instance, Cluster 2 suggests a connection between accidents among workers aged 61 to 80, with low levels of education, who work independently in the forestry, agriculture, and fishing sectors. This association hints at a certain informality, with older workers with limited access to education working independently in a sector often associated with family production. Cluster 5, on the other hand, highlights a unique attribute related to gender, indicating female workers with a high level of education, working as technicians or in administrative services, and experiencing accidents that affect the entire body. These non-obvious relationships underscore the need for further investigation to extract more detailed information and develop a comprehensive interpretation of these associations. Cluster 6 provides information on accidents involving public servants, both statutory and formal contract categories, in science and arts positions, as well as with a complete higher education degree.

Cluster 7 was formed in 6 indexes: ET, DI, HI, AT, MDI, and WCT. All indexes were calculated and divided into five categories according to their minimum and maximum. It is possible to note that, in all cases, Cluster 7 encompasses the index’s last and highest range of values. The highest range of values of the ET index is from 27.7 to 35.6; of the DI, it is from 27.6 to 35.5; of the HI, it is from 144 to 187; of the AT index, it is from 34.8 to 44.5; of the MDI, it is from 28.5 to 35.5; and of the WCT Index, it is from 35.6 to 46.4. These values are within the critical limits or exceed the limits of the indexes, indicating a greater danger for this cluster [39]. Associated with this risk are two regions of Brazil, the attributes UF: A and UF: B, which refer to the North and Northeast Regions. These results agree with those previously found, associating the highest range of heat stress values with the North and Northeast Regions [21]. The North and Northeast Regions do not have any particularity that could justify this behavior besides the climatic effect. A previous exploration of this same database indicated that these two regions are the two with the lowest number of accidents, with the South and Southeast Regions being the ones with the highest rates, as these are the most populated [40]. However, these regions are among the ones with the highest probability of becoming hotter over the coming years [41].

Cluster 3 encompasses numerous attributes due to the proximity of the points. However, for a clear interpretation, the focus should be on each of the index values and the points closest to them, disregarding distant points even if they fall within Cluster 3. This approach will help in extracting meaningful insights from the data.

Starting with the smallest range of values for each index. For the ET index, for example, the minimum range varies from −3.64 to 4.23, and the closest attributes are PC: A and PC: F, which represent two affected body parts, the eyes and hands, respectively. This characteristic is repeated for the DI, ETW, HX, AT, MDI, and WCT Indexes. In the HX Index, there is also proximity to the Oc: I attribute, which represents workers’ occupation in producing industrial goods and services. The HI differed the most from the others. The smallest range of HI values is from −26.8 to 16.1 and is close to the PC: G attributes, representing the upper limb; Rc: C, which represents Asians; and EV: B, which represents the evolution of the case as a temporary disability.

The second smallest range of values showed similar behavior. The ET, DI, ETW, AT, MDI, and WCT Indexes indicated proximity to the Lo: A and UF: D attributes, representing the accident location such as the contractor’s facilities and the Southern Region of Brazil. For the ETW and MDI Indexes, it is possible to associate the attribute Oc: I with them, which represents workers in producing industrial goods and services. The HI, in turn, approximates this value range to the attributes Es: E, representing the level of education of complete elementary school; CAT: B, indicating the non-opening of CAT; and the occupations Oc: H and Oc: J, which represent workers in the production of industrial goods and services and workers in repair and maintenance services, respectively.

The HX index values are more spread out in Cluster 3, so it is possible to associate more attributes with each of them. For the second smallest range of values, the following are associated, Lo: A and UF: D, as in the previous clusters, but it is also possible to include Rc: A, which indicates that the race of the injured worker is White; ST: A, which indicates that the worker is a registered employee with a formal employment contract; UF: C, indicating the Southeast Region of Brazil; CAT: A, indicating that a CAT was opened; EV: A and EV: G, indicating the evolution of the case with cure or others, respectively; Id: B, representing the age group from 18 to 25 years; PC: K, with the affected body part having a value of “others”; and Es: G, representing the education level of complete high school.

The third range of values repeats the pattern. The ET, DI, ETW, AT, MDI, and WCT Indexes are similar. The associated attributes are Rc: A, which represents White workers; EV: A and EV: G, which represents that the case evolved to the cure of the victim or “others”, respectively; Id: B, representing the age range from 18 to 25 years; Es: F and Es: G, representing that the level of education is incomplete and complete high school, respectively; ST: A, which indicates that the worker is registered; UF: C, which indicates that the worker is in the Southeast Region of Brazil; PC: I and PC: K, which indicate that the affected body part was the feet or “others”. These values are similar to the previous range of values of the HX index. The HX index, in turn, for this range of values presents as an association the attributes Rc: B and Rc: C, which indicates that the worker’s race is Black or Asian, respectively; Id: A and Id: D, which represent the age ranges of under 18 years and 41 to 60 years, respectively; Sx: M, which represents male workers; Es: E, which indicates that the level of education is complete elementary school; PC: B, PC: D, PC: E and PC: I, which indicate that the affected body parts encompassed the head, chest, abdomen, and feet, respectively; CAT: B, which indicates that the CAT was not opened; Oc: J, which represents workers in repair and maintenance services; and TY: A, indicating that the accidents were of the typical type. Finally, the HI shows the association of this range of values with the attributes Rc: B, which indicates Black workers; Sx: M, indicating male workers; Id: C, which represents the age range from 26 to 40 years; EV: A and EV: G, which represent that the case evolved to cure or “others”, respectively; and PC: I, indicating that the foot was the affected body part.

The fourth range of values encompasses the second highest values of the indexes and may already present a risk depending on the index in question. The attributes that appear associated with the ET, DI, ETW, AT, MDI, and WCT Indexes are Id: D, which represents the age group from 41 to 60; Rc: C and Rc: D, which represent the Asian and Brown races; UF: E, representing the Central-West Region; ST: H and ST: K, representing both the situation of the worker as an employer and a temporary worker, respectively; PC: B, PC: D, and PC: E, representing that the affected body parts were the head, thorax, and abdomen, respectively; and EV: B and EV: E, indicating that the accident evolved either to temporary disability or to death due to a severe work accident. For the HX index, the associated attributes are ST: H, ST: I, and ST: L, which indicate that the worker was a temporary worker, cooperative worker, or “other”; Rc: D, which indicates the Brown race; UF: E, indicating the Central-West Region; EV: E and EV: F, which indicate that the case evolved into death due to a severe work-related accident and death from other causes; and ST: H, representing a temporary worker. As for the HI, this range of values is related to the attributes PC: B, PC: D, and PC: E, which indicate that the affected body parts were the head, thorax, and abdomen, respectively, and Rc: D, representing the Brown race. The fourth range of values had the most significant agreement with regard to the results among all indexes.

Finally, the highest value range of the ET, DI, HI, AT, MDI, and WCT Indexes was already discussed for Cluster 7. For the ETW Index, this range is associated with the attributes UF: A and UF: B, which represent the North and Northeast Regions of Brazil; ST: I, and ST: L, which indicate that the worker is a cooperative member or “other”; and EV: F, which represents that the case evolved into death from other causes. For the HX index, the highest value range is associated with ST: B and ST: G, which indicate that the worker was an unregistered employee or unemployed, respectively; EV: D, which indicates that the case evolved into permanent total disability; and Rc: E, which represents that the workers were of the Indigenous race.

## 4. Discussion

With the results obtained, we can raise some discussions. The application of MCA is an efficient way to visualize the distribution of variables present in a database and graphically evaluate the relationship between them. For the present study, the visual aspect of MCA served not only to explore the relationship between variables related to work accidents and heat stress indexes, pointing out essential relationships between values that exceed the limits of tolerance to thermal risks with characteristics of the injured workers: its application was also helpful in the sense of comparing different heat stress indexes. By placing the graphs side by side, the differences and similarities of the results between the indexes become evident.

The clusters created can be classified into two types. The first type involves accidents unrelated to heat stress: Clusters 1, 2, 4, 5, and 6. It is possible to argue that the variables that classify these clusters are not affected by climate variables and remain the same among all indexes. We can validate this finding by comparing it with previous results, in which there are also five clusters without heat stress indexes whose attributes are mostly the same as those presented in this study [21]. Slight differences between our study and the previous results may have occurred due to the data update, which added records from 2020 to 2023.

Of the eight indexes, seven had very similar results. The five value ranges of the TE, DI, HX, TEV, AT, MDI, and WCT Indexes showed similar behaviors, associating the same variables most of the time, with only a few slight variations in some indexes. The HI showed the most significant difference from the other indexes, showing the various behaviors of the relationships between the attributes that were very different from those of the other indexes. This disparity of the HI can be explained by the fact that it is best applied to temperatures above 27 °C and relative humidity above 40%, a fact that was not considered for the present study because it would reduce the database, differentiating it from the other indexes. This similarity between the TE, DI, HX, TEV, AT, MDI, and WCT Indexes allows us to conclude that there is no difference in using one of these indexes in opposition to the others in the investigation of the risk of heat stress in the occurrence of work accidents, as all seven indexes reached similar conclusions. The HI is the only index that differed, but its use requires a limitation to the range of valid temperature and humidity to be applied.

This article contributes to the literature in two main ways. It expands the research on the risks of high temperatures on workers’ health through a study that explores historical data on work accidents in Brazil. This type of research is essential to demonstrate, through actual data, the impacts of climate change and the need to mitigate these risks that are increasingly present in the reality of the working population. The second point concerns the distinction between the heat stress indexes. In the literature, no specific factor defines the quality of an index in relation to others; there is only the tendency for use by other researchers, making some indexes more popular than others, and even the use of these indexes in national or international norms and standards. This study confirms that the use of different indexes can generate similar results, regardless of the differences how they are calculated. It is important to emphasize that, just like the HI, which deviated from the others, it is necessary to investigate whether each index has its own limitations and requirements to ensure a good result. It is also important to highlight a limitation of this study. The conclusions reached concerning the behavior of the indexes are exclusive to the database used in this study. A different database with different attributes may present different results that point to significant differences between the indexes. This underscores the need for caution when interpreting and applying this study’s findings. However, in the climate and occupational health context, it is imperative to consider regional data and understand the meteorological, social, and economic varieties specific to that location.

We have four suggestions for future studies. The most straightforward approach is to adapt the database, reducing it to consider only records with temperatures above 27 °C and 40% of relative humidity, as required by the HX Index, and to compare it with others. The second suggestion regards the use of the most popular heat stress index, the WBGT index. The model developed by Liljegren et al. (2008) includes air temperature, relative humidity, solar radiation, and wind speed for its calculation, and it is considered one of the most reliable models for calculating this index [42,43]. However, we did not include this index in our present study because the solar radiation data provided by the weather stations was not precise and presented problems with consistency. Other research that can obtain access to different meteorological data sources could use this index and fill in this gap.

The third suggestion is to expand this comparison and use indexes to investigate occupational thermal risks beyond statistical techniques, implementing computational tools, such as data mining and machine learning, to deepen the exploration of this information and extract the maximum knowledge about this problem. The fourth suggestion is to segment the database into smaller sets focused on specific characteristics, such as a dataset of occupational accidents of a single occupation comprising an industrial sector. We believe that a more accurate study could be achieved if our database was composed only of heat-related injuries, making the connection to the climate more direct. However, the Sinan database, which is the primary source of data for this study, or any other Brazilian database that records this type of information, does not include any records that could make this connection. Therefore, to analyze the occupational health in Brazil, we must deal with data regarding every type of work incident, making the relationship to the climate more challenging to discover.

## Figures and Tables

**Table 1:** Summary of the indexes used, the environmental factors used in their calculations, a symbol to differentiate them, and their range of values.

Heat Stress Index	Environmental Factors	Symbol	Range of Values
ET	Air temperature and relative humidity	Blue Circle	I: −3.64 to 4.23 II: 4.23 to 12.1 III: 12.1 to 19.9 IV: 19.9 to 27.7 V: 27.7 to 35.6
DI	Air temperature and relative humidity	Green Pentagon	I: −3.64 to 4.21 II: 4.21 to 12 III: 12 to 19.8 IV: 19.8 to 27.6 V: 27.6 to 35.5
HI	Air temperature and elative humidity	Yellow Square	I: −26.8 to 16.1 II: 16.1 to 58.8 III: 58.8 to 102 IV: 102 to 144 V: 144 to 187
ETW	Air temperature, relative humidity, and wind speed	Pink Hexagon	I: −19.2 to −4.58 II: −4.58 to 9.96 III: 9.96 to 24.5 IV: 24.5 to 39.1 V: 39.1 to 53.7
HX	Air temperature and relative humidity	Cyan Diamond	I: −6.62 to 7.12 II: 7.12 to 20.8 III: 20.8 to 34.5 IV: 34.5 to 48.2 V: 48.2 to 61.9
AT	Air temperature and relative humidity	Orange Arrow	I: −3.61 to 6.02 II: 6.02 to 15.6 III: 15.06 to 25.5 IV: 25.2 to 34.8 V: 34.8 to 44.5
MDI	Air temperature, relative humidity, and wind speed	Brown Heart	I: 0.6 to 7.6 II: 7.6 to 14.6 III: 14.6 to 21.5 IV: 21.5 to 28.5 V: 28.5 to 35.5
WCT	Air temperature and wind speed	Black Moon	I: −7.64 to 3.21 II: 3.21 to 14 III: 14 to 24.8 IV: 24.8 to 35.6 V: 35.6 to 46.4

**Table 2:** Summary of all accident record variables and their respective possible values.

Acronym	Variable and Its Value	Acronym	Variable and Its Value
**UF**	**Region** A: North B: Northeast C: Southeast D: South E: Midwest	**ST**	**Employment status** A: Registered employee B: Unregistered employee C: Self-employed D: Statutory public server E: Registered public server F: Retired G: Unemployed H: Temporary work I: Cooperative worker J: Freelancer K: Employer L: Others
**Sx**	**Gender** F: Female M: Male	**Ti**	**Type** A: Typical B: Commuting
**Id**	**Age** A: Below 18 years B: 18 to 25 years C: 26 to 40 years D: 41 to 60 years E: 61 to 80 years F: Above 81 years	**Lo**	**Place of Accident** A: Contractor’s premises B: Public thoroughfare C: Third part’s installations D: Own home
**Rc**	**Race** A: White B: Black C: Yellow D: Parda E: Indigenous	**PC**	**Body part affected** A: Eye B: Head C: Neck D: Chest E: Abdomen F: Hand G: Upper limbs H: Lower limbs I: Feet J: Whole body K: Other
**Es**	**Education degree** A: Illiterate B: Incomplete 1st Elementary School C: Complete 1st Elementary School D: Incomplete 2nd Elementary School E: Complete 2nd Elementary School F: Incomplete High School G: Complete High School H: Incomplete Higher Education I: Complete Higher Education	**Ev**	**Evolution** A: Recovery B: Temporary incapacity C: Permanent partial disability D: Permanent total disability E: Death from serious work accident F: Death from other causes G: Others
**Oc**	**Occupation** A: Military forces, police officers, and firefighters B: Members of the public, leaders of public interest companies, managers C: Sciences and arts professionals D: Mid-level technicians E: Workers of administrative services F: Services, retailers and sales workers G: Agricultural, forestry and fishery workers H: Discrete production of industrial goods and services workers I: Continuous production of industrial goods and services workers J: Repair and maintenance services workers	**CAT**	**CAT Report** A: Yes B: No C: Not applicable

**Table 3:** Values of each variable in Clusters 1, 2, 4, 5, and 6.

Cluster	Variable	Value
1	Es: F	Employment status: retired
	Id: F	Age group: over 81 years
	Lo: D	Accident location: own home
2	WS: C	Employment status: self-employed
	Oc: G	Occupation: agricultural, forestry and fishing workers
	Es: B	Education level: 1st to 4th grade of elementary school
	Id: E	Age: 61 to 80 years
4	TY: B	Accident type: commuting
	Lo: B	Accident location: public roads
5	PC: J	Body part: entire body
	Es: H	Education level: incomplete higher education
	Oc: D	Occupation: mid-level technicians
	Sx: F	Sex: female
	Oc: E	Occupation: administrative services
6	Es: D	Employment status: statutory public servant
	Es: E	Employment status: formal contract public servant
	Oc: G	Occupation: professionals in the sciences and arts
	Es: I	Education level: complete higher education

## Data Availability

Research data is not available as it is being refined and prepared into an usable database, it is a working in progress and include some privacy restrictions.
